# Dynamic Tracking Algorithm for Time-Varying Neuronal Network Connectivity using Wide-Field Optical Image Video Sequences

**DOI:** 10.1038/s41598-020-59227-5

**Published:** 2020-02-13

**Authors:** Carlos Renteria, Yuan-Zhi Liu, Eric J. Chaney, Ronit Barkalifa, Parijat Sengupta, Stephen A. Boppart

**Affiliations:** 1 Beckman Institute for Advanced Science and Technology, Urbana, USA; 2 Department of Bioengineering, Urbana, USA; 3 Department of Electrical and Computer Engineering, Urbana, USA; 4 Neuroscience Program, Urbana, USA; 5 Carle Illinois College of Medicine, University of Illinois at Urbana-Champaign, Champaign, USA

**Keywords:** Data processing, Image processing, Computational science

## Abstract

Propagation of signals between neurons and brain regions provides information about the functional properties of neural networks, and thus information transfer. Advances in optical imaging and statistical analyses of acquired optical signals have yielded various metrics for inferring neural connectivity, and hence for mapping signal intercorrelation. However, a single coefficient is traditionally derived to classify the connection strength between two cells, ignoring the fact that neural systems are inherently time-variant systems. To overcome these limitations, we utilized a time-varying Pearson’s correlation coefficient, spike-sorting, wavelet transform, and wavelet coherence of calcium transients from DIV 12–15 hippocampal neurons from GCaMP6s mice after applying various concentrations of glutamate. Results provide a comprehensive overview of resulting firing patterns, network connectivity, signal directionality, and network properties. Together, these metrics provide a more comprehensive and robust method of analyzing transient neural signals, and enable future investigations for tracking the effects of different stimuli on network properties.

## Introduction

In the past decade, substantial efforts aimed to assess functional associations between brain regions and external stimuli. The advent of genetically encoded calcium indicators (GECIs) has provided an all-optical approach for this assessment that has been widely adopted for neuroscience research^1–9^. In addition, sophisticated optical imaging and optogenetic photostimulation systems have been developed and used to generate vast amounts of data, all of which provide some form of assessment of optical activity associated with neural functioning^10–23^. With the advent of these technologies comes the increased volume of generated data and the increased complexity of analysis. Thus, determining how neuronal networks propagate signals and the degree of neuronal interconnectivity based on their firing patterns remains a tedious task. Many groups have developed algorithms that aim to determine this interconnectivity based on the firing patterns in neural networks^24–28^, many based on the Hebbian model that synchronous neural firing coincides strongly with neural connectivity. These algorithms typically fall into two categories: model-based approaches and model-free approaches. Model-based approaches take into consideration the kinetics of the GECIs in a biophysical model for their algorithms^29,30^. Alternatively, model-free approaches base their analyses on the signals acquired by the cell bodies, and perform statistical analyses independent of any physical model^31–36^. As such, these approaches tend to be more easily implementable, and require less computational power.

Traditionally, algorithms developed for monitoring neuronal connectivity are validated using computational models of network dynamics^28,37^. These models use known synaptic weights to construct artificial networks of cellular dynamics, and if the network topology is known, provide a ground-truth assessment of the validity of the network analyses. However, ground-truth is typically unknown in most neural cell culture models, and especially in intact brain tissue. Thus, forming a basis for validating the transfer of information between neurons has remained a bottleneck in network assessment. The complexity of functional neuronal networks far outweighs even the most robust models, and experimental ground-truths need to be used as a method for verifying algorithms. Most algorithms also lack the ability to infer directionality of signal propagation without dramatically increasing the sampling rate^25^. Some connectivity metrics, such as the Grainger Causality, have been heavily debated over concerns of their accuracy, and whether the information obtained from these analyses is adequately interpreted^28,38–40^.

**Figure 1:**
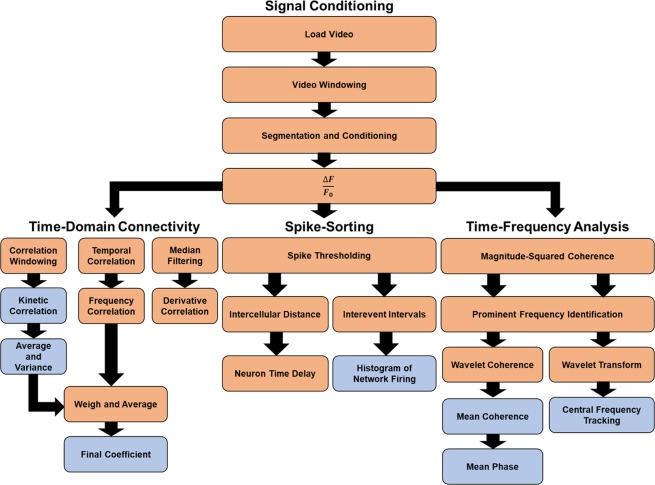
Flow-chart of the network analysis algorithm and the parameters extracted from the imaging data. The video sequence (AVI format) is first loaded into the Matlab workspace and segmented temporally into tenths to reduce memory usage. The isolated signal from each individual neuron in the video sequence is then processed for fluorescence normalization, and then processed to acquire time-domain connectivity parameters, network firing properties, and coherence analyses for time-frequency metrics.

In this study, we propose not only a new algorithm to assess network connectivity (Fig. 1), but also a framework by which validation of network responses can be obtained. The algorithm itself takes a model-free approach, integrating classical techniques for assessment of network topology. The algorithm uses a temporal Pearson’s correlation coefficient, as well as wavelet coherence, to determine the degree to which neurons are interconnected based on the time-frequency information obtained from these signals. The Pearson’s coefficient is a widely adopted and well-known measure of correlation, and is also very easy to calculate. It has also been demonstrated to be very effective at inferring correlation than many other more advanced techniques^28^. Therefore, the metric is easily interpretable, making it a strong candidate for preliminary analysis of network correlation. Wavelet coherence, though less commonly utilized, provides added dimensionality to the signal content, quantifies correlation strength, and through the phase information obtained from the transform, can also be used to quantify delays between signals, and consequently, directionality^41^. This proves to be a superior metric that overcomes each of the aforementioned limitations, and increases the breadth of useful information available to the researcher.

Furthermore, this algorithm segments the calcium transients extracted from the cell bodies of interests into discrete time-windows to assess temporal changes in connectivity throughout the course of the experiments. Further windowing is performed along these discrete windows to obtain a localized assessment of neuronal connectivity as well, rather than basing analyses only on a correlation coefficient extracted throughout the entire time frame. Recent work in neural signal processing has demonstrated the utility of dynamic connectivity measurements for characterization of mental disorders using fMRI^42^. Results from experiments performed on cells exposed to glutamate to elicit neuronal activity demonstrated the capability of the algorithm to determine and pinpoint changes in connectivity during chemical activation. We demonstrate this algorithm as a method for detecting changes in network activity over time by temporally assessing network dynamics after the application of glutamate, specifically analyzing its effects on cellular function and network stability in culture.

## Results

### Time-varying connectivity coefficients reveal dynamic nature of connectivity

**Figure 2:**
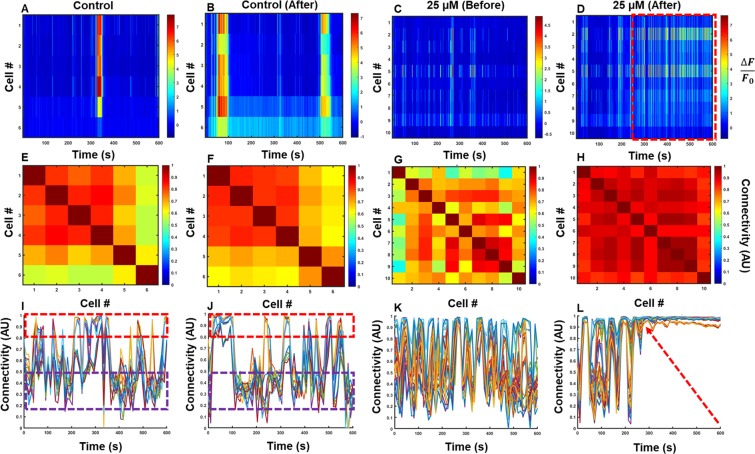
Monitored, time-varying, multicellular firing activity and connectivity. (**A–D**) Cellular firing maps, (**E–H**) connectivity plots, and (**I–L**) plots of the time-varying connectivity for all pairs of cells before and after application of glutamate or cell-culture media. Time-series calcium traces are plotted for each cell in an FOV (**A,C**) before and (**B,D**) after application of (**A,B**) cell culture media and (**C,D**) 25 µM glutamate. The red dashed box in (**D**) is highlighting the region of increased cellular firing frequency, which coincides with the increased global connectivity (**L**, arrow). The red and purple dashed boxes in (**I**,**J**) are representative of high connectivity (above 0.8) and low connectivity (below 0.5), respectively.

**Figure 3:**
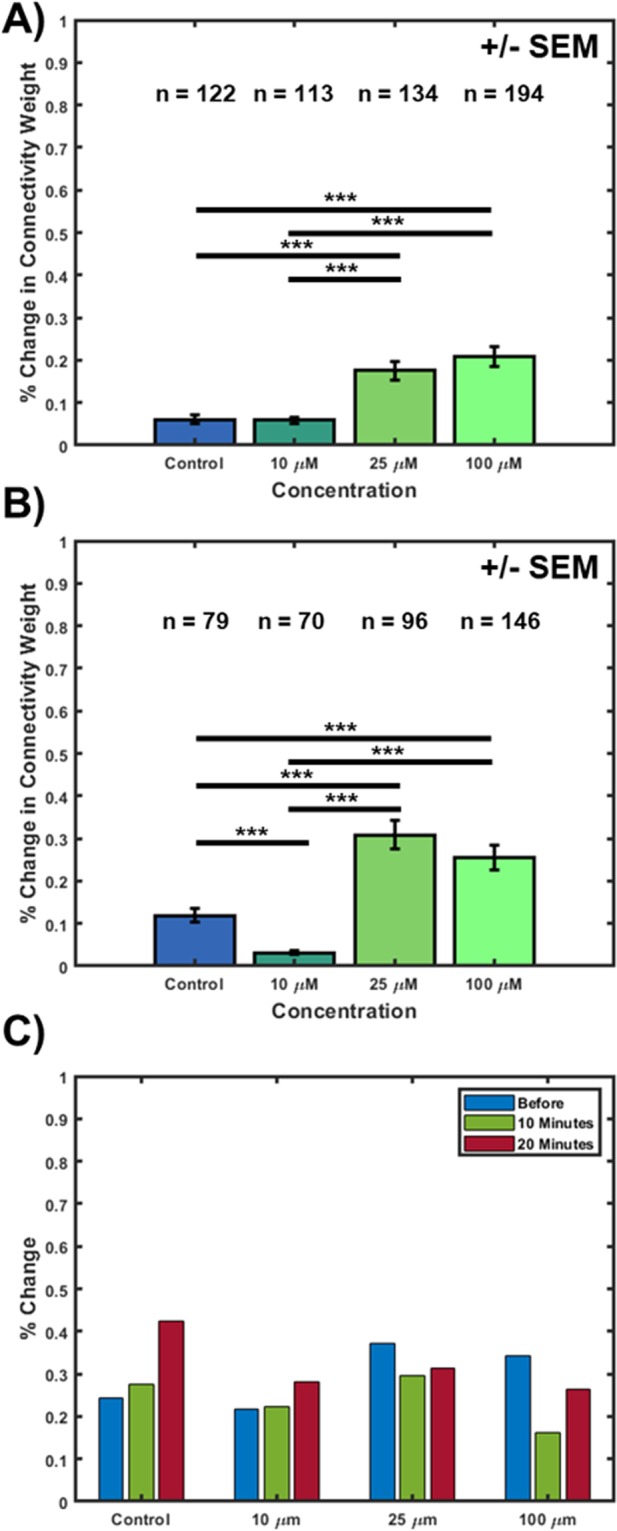
Measured connectivity change based on varying concentrations of glutamate after (**A**) 10 minutes, and (**B**) 20 minutes. (**C**) Temporal change for all experimental conditions within each imaging window. Sample size (n = #) is the number of cell pairs for each experimental condition—all from different cultures. ***represents p < 0.005.

In this work, we implemented a time-varying Pearson’s correlation coefficient to analyze the calcium transients acquired from cultured, hippocampal neurons. Hippocampal neurons were isolated from transgenic mice expressing GCaMP6s, and cultured for 12–15 days before imaging (Supplementary Figure S1). Glutamate at various concentrations was used to elicit a response from the culture, due to its excitatory effects in neurons. The time-varying coefficients illustrate dynamic changes of neural communication strength based on neural firing. The dynamic nature after application of glutamate is illustrated in Fig. 2. Representative calcium transients, corresponding connectivity matrices, and time-varying connectivity coefficients illustrate the effect of chemical and control conditions. A representative plot of the coefficient changes over time illustrates the fluctuations that are present in neural systems. In contrast to general connectivity matrices which provide a single coefficient based on the entire temporal trace, dynamic neural connectivity demonstrates fluctuations in connectivity. More uniform temporal connectivity is shown in Fig. 2L, after excitation with 25 µM glutamate. Interestingly, this trace also highlights the instance when this uniformity occurred—roughly 270 seconds after the start of imaging. The control culture (Fig. 2I,J) continues to demonstrate fluctuations after initial introduction of culture media, suggesting that global excitation with glutamate promotes synchronous firing of neurons, and consequently increased connectivity due to this simultaneous firing. These results are repeatable (Supplementary Figure S2) in all experimental conditions, demonstrating uniform connectivity increases for all applications of glutamate. For the 10 µM, 25 µM, and 100 µM concentrations, there is a large increase in global connectivity that is absent in the control cultures, further confirming this phenomenon in all experimental conditions. The control conditions also show significantly larger mean percent difference than all other glutamate-induced conditions over time, especially after 20 minutes (Fig. 3C), validating that this result is consistent across different cultures. Initially there appears to be little difference in fluctuations, but over time, this variation increases in comparison to the glutamate-induced conditions. This is attributed strongly to saturation of the connectivity values for glutamate-induced cases, as exemplified in Fig. 2L. This, in addition to a lack of reduction in the connectivity values for the glutamate-induced cases, results in decreased fluctuations in connectivity relative to the controls.

These results indicate that the neural connectivity inherently varies over time, and that chemical induction with glutamate reduces this variability. This supports other research that demonstrates inherent fluctuations involved in underlying neural mechanisms, such as mobility in healthy and diseased states^43^, and that this time-varying approach could identify different states of connectivity using the temporal knowledge of connectivity and knowledge of the stimulus^42^. The literature demonstrates that by using fMRI, there are notable differences in temporal connectivity dynamics based on whatever external stimulus is applied. Interestingly, dynamic network connectivity is becoming increasingly popular in fMRI studies, but its application for connectomic studies in microscopy systems has been limited. We propose that within these fluctuations, there are other mechanisms that can be used to discriminate functional states for myriads of electrochemical perturbations in neural systems. Furthermore, perturbations by optogenetic stimuli and tailoring of incident light pulses could also be probed with time-varying methods to see how this modulates connectivity, temporally. This combination of tools would provide a powerful method by which an underlying, dynamic process could be discovered and monitored, and consequently, a stronger understanding of neuronal connectomes could be achieved.

### Glutamate induces changes in network connectivity

Observing that there were clear fluctuations in cellular connectivity that were disrupted by glutamate uptake, we wanted to determine whether there would be notable, aggregate differences in connectivity resulting from different concentrations. To monitor aggregate differences in connectivity following administration of glutamate, the percent-change of connectivity values from baseline to 10 or 20 minutes was calculated using the generalized, “final” connectivity coefficient (Fig. 1). For tracking more dynamic temporal changes, the mean percent-change at one-minute intervals was calculated and plotted before, 10 minutes after, and 20 minutes after glutamate administration. Figure 3 shows the percent-change progression of these connectivity values as a result of each concentration of glutamate after 10 minutes and 20 minutes post-administration. Significant differences in connectivity exist between the control and the 25 µM and 100 µM conditions. This same difference is seen between 10 µM and the two higher concentrations, after 10 minutes. This same trend continues after 20 minutes, but with significant differences also noted between the control and the 10 µM condition, and no significant difference between both higher concentrations at any time point. Figure 3A shows that for the control and 10 µM conditions, there is very little percent-change in connectivity compared to baseline at 10 and 20 minutes. In contrast, for both the 25 µM and 100 µM conditions, overall connectivity changed dramatically. After 20 minutes (Fig. 3B), the control and 10 µM conditions also showed statistically significant differences. For the 10 µM condition, the connectivity approached the calculated baseline connectivity values, which did not occur for each of the other experimental conditions.

It is believed that there may be a biphasic effect, where concentrations near 10 µM may perturb (increase) neural connectivity for brief periods of time, and then return to regular connectivity values shortly thereafter. Indeed, although glutamate is pivotal for establishing neural connectivity through long-term potentiation (LTP), higher concentrations elicit a neurotoxic response, which ultimately results in cell death^44,45^. Neurons have many mechanisms to regulate glutamate and other neurotransmitter release by storage and release^46^ but only to a certain level. Previous literature has shown significant toxicity effects above 10 µM^44^, which becomes especially profound beyond 100 µM, and over time, this toxicity increases. The same study^44^ showed an exponential increase in acute and chronic cytotoxicity after the administration of 200 µM glutamate at DIV 14. Measurements in lactate dehydrogenase (LDH) were used to assess toxicity measurements. The authors suggest that different cytotoxic pathways are reached at different times after exposure to toxic levels of glutamate, which we believe is occurring in the presented work. In contrast, however, other studies demonstrate that these higher concentrations, *in vitro*, promote recovery of electrical activity^47^. From these studies, we infer that, on average, concentrations around 25 µM may be the threshold for neurotoxicity in cultured neurons, and that over time, lower concentrations are regulated through inherent neural mechanisms, such as glial transmission or otherwise^48^. In contrast, lower or higher concentrations would result in either dramatic changes in connectivity (higher dosages), or no changes to the inherent variability in connectivity (low to no dosage), respectively. These mechanisms need to be investigated further to study this effect, and see if there is indeed an underlying mechanism that causes excito-toxicity at these high concentrations, while promoting healthy neural function at lower concentrations.

Additionally, variations in connectivity over time were studied over the course of each recording. Low variations in weights are seen during the initial ten-minute recording (Fig. 2I), indicative of their consistency during this time. Regions of strong connectivity are outlined by the red dashed box, and those of weaker connectivity by the purple dashed box to demonstrate the fluctuations present in the data. Following chemical stimulation by glutamate, significant modulations in the variability of weights that correspond to increased fluctuations in cellular firing patterns were observed initially, followed by stabilization of activity during post-chemical stimulation, and then new corresponding weights. The control cultures show larger variations in connectivity over time after 10 and 20 minutes, in comparison to the other cultures. The 10 µM conditions show very similar trends, but are much less pronounced than the control. In both cases, the variation in network connectivity increases over time. In contrast, variability drops for the 25 µM and 100 µM conditions, and fluctuations increase again afterwards—demonstrated by the dramatic increase in global connectivity. The 10 µM condition shows gradual fluctuations, but is comparable to the control culture initially. In all cases, over the course of 20 minutes, there is less variability than in the control sample, demonstrating a decrease in fluctuations in neural connectivity after applying glutamate. The 25 µM and 100 µM conditions show a sudden decrease in fluctuations, which begins to rise again after 20 minutes. These oscillations can also be more readily visualized in Supplementary Figure S2, which shows a temporal plot that illustrates the variation in connectivity weights over time.

### Wavelet analyses show increased coherence in glutamate-treated cultures, highlights frequency content

Given the inherently time-variant nature of neural firing and connectivity, we believed there should be a more comprehensive method for analyzing the dynamic nature in neural systems. The wavelet transform has become increasingly adopted for monitoring dynamic systems, and wavelet coherence has more recently been adopted as an assessment of determining causality and correlation of signals, while discriminating between frequencies that may or may not contribute to these similarities^49^. To our surprise, this method has not been widely utilized in calcium imaging for establishing coherence between two signals of interest, so we adopted it in this work to investigate what patterns could be acquired from these signals. By taking a wavelet transform of the acquired signals, the spectral content was extracted and quantified over time. After the application of 25 µM glutamate (Supplementary Figure S3, B–C), there was a gradual convergence of the neural firing frequency toward 0.3 Hz, where higher intensities (those regions in red on the plot) are more indicative of increased signal strength at that frequency band. Whereas in the unperturbed and control samples (Figure S3, E–F), there is a broad spectrum of signals, and the bandwidth converges dramatically after the application of glutamate. This, functionally results in the ability to determine a causal relationship between the glutamate stimulus and this convergence, illustrating a powerful benefit of wavelet analysis for analyzing neural signals. Prior to this stimulus, and in the control cultures, a much broader spectrum of signal frequencies was present, due to the variable nature of the firing patterns of the neurons. The increased firing frequency and increased uniformity in signals resulted in both an increase in apparent global network connectivity and more rapid synchronous firing as a result of the application of glutamate.

**Figure 4:**
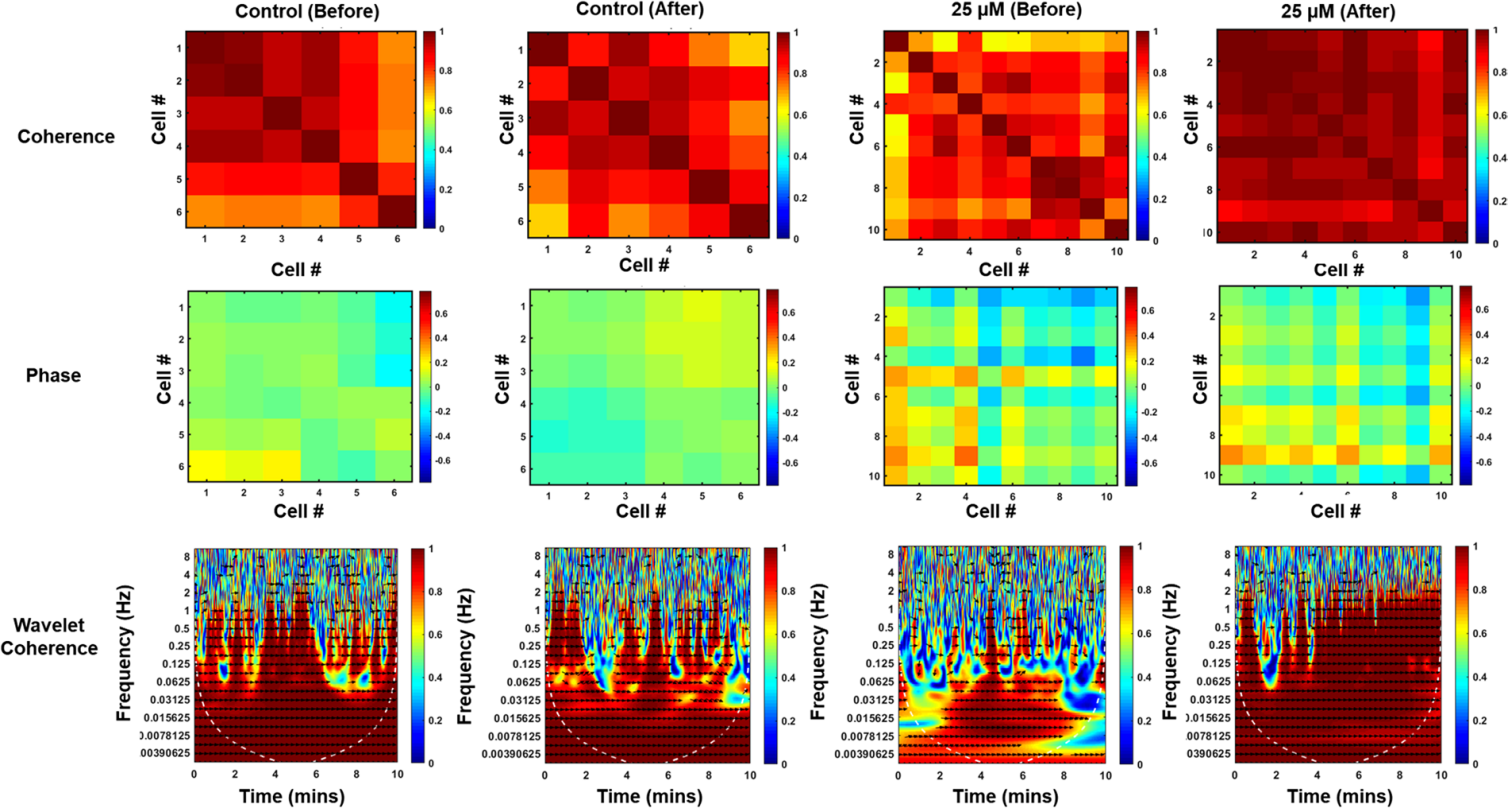
Representative plots of coherence matrices, phase matrices, and wavelet-coherence transforms that were utilized in this study. There are similarities in the relative intensities and patterns that correlate with the data shown in Fig. 2.

Wavelet coherence, on the other hand, provides similar information to the time-varying connectivity coefficients. Figure 4 emphasizes both the coherence and phase information acquired from the signals between two cells. This information is easily quantified and provides information not just about the strength of connectivity based on signals, but also the directionality using the phase information. The coherence and connectivity metrics match very well, supporting that both metrics give complementary information. The wavelet coherence, however, also provides the benefit of showing the phase lag between two signals. Thus, in addition to signal connection strength, the directionality of signal propagation can also be obtained. This provides an additional benefit that is otherwise not present even with the time-varying Pearson’s correlation approach, quantifying phase delays between signals. Similar to the time-varying correlation coefficient, it is readily apparent after the application of glutamate that there is a universal increase in network connectivity, as well as a universal increase in coherence across all frequencies. This change is not seen as dramatically in the control condition. There was also a different shift in the phase information relative to the control sample. This translates to a difference in signal propagation in the network, which would otherwise not be revealed with traditional methods. The wavelet coherence approach provides similar information about connection strength as the Pearson’s correlation coefficient (Supplementary Figure S4, A) by illustrating a regression of all the time-varying, time-averaged connectivity data with the time-averaged coherence data, to show a linear relationship between them (R = 0.7367). These results, therefore, demonstrate that the wavelet approach to analyze temporal data is simple, comprehensive, and robust, providing strong levels of detail with regards to the temporal dynamics of neural signals. Consequently, the added frequency and phase information add to the breadth of information that can readily be obtained for analyzing the dynamic nature of neural systems, as discussed in the time-varying connectivity section. Also demonstrated is the strong association between this firing frequency and network connectivity (Supplementary Figure S4, B). Previous studies have demonstrated that increases in functional connectivity are strongly associated with increasing firing frequencies^50^, attributed to the temporal proximity of neighboring spikes at higher frequencies. As such, these variables are highly intertwined, making it difficult to discriminate connectivity independent of firing rate. The results highlighted here further validate the presence of this relationship, while an ability to discriminate these factors remains elusive. Highly controlled experiments of optogenetic inhibition along the synapses between neurons may be sufficient to discriminate between these variables, however, the inherent link between these variables cannot otherwise be easily discriminated computationally. Together, these metrics provide a comprehensive analysis for dynamic network activity using optical imaging, opening the door to powerful applications for neurochemical reactions, connectomics, drug discovery, and a large number of other applications. Neural systems are inherently time-variant based on external stimuli, so using this time-varying approach for monitoring network properties serves as a useful computational tool for neuroscience research.

### Network firing properties illustrate changes to all conditions, independent of connectivity changes

**Figure 5:**
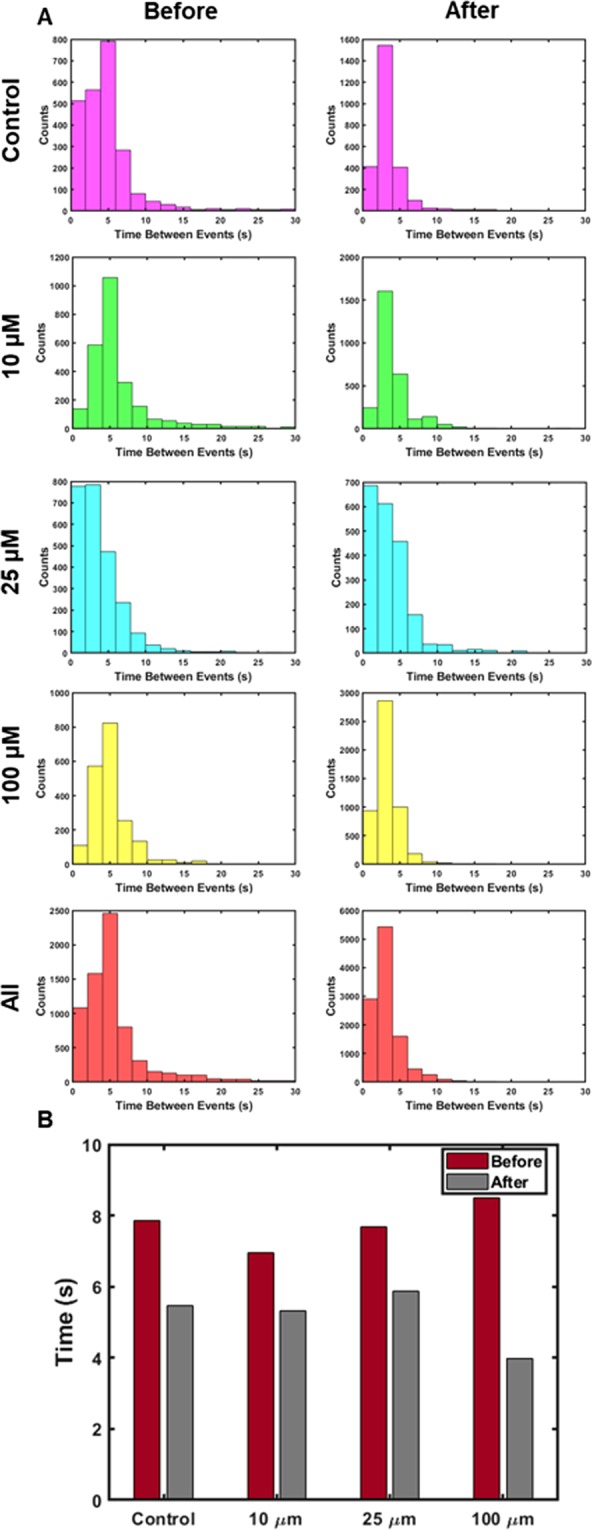
Histograms of inter-event intervals for each concentration condition before and after application of the chemical stimulus. (**A**) The inter-event interval histograms for each individual experimental condition, including all conditions clustered together. (**B**) Mean inter-event intervals for each individual experimental condition, before and after glutamate administration.

The firing patterns of individual neurons in the cultured networks were also investigated in aggregate. This required the identification of more prominent calcium traces, and determining the temporal differences between spikes. In this study, significant calcium spikes were segregated by applying a threshold two standard deviations above the mean fluorescence of the calcium traces. Peaks within 0.5 seconds of one another were not counted as separate spikes, and subsequent spikes had to be separated by at least 0.5 seconds to be considered separate spikes. After thresholding by time and amplitude, the temporal difference between subsequent calcium events was calculated, and this was then used as the inter-event interval. These intervals were then used to monitor the firing patterns of individual neurons. The inter-event intervals calculated in this study followed a Poisson distribution, with decreased means after all chemical stimuli conditions (Fig. 5). Each condition demonstrated the same histogram shape, with the primary difference being the mean inter-event intervals. In each case, there was a decrease in the mean inter-event interval, although there was no overall change to the distribution. The firing rate decrease was most prominent for the 100 µM condition, which was expected considering the excitatory nature of glutamate. However, all conditions demonstrated this increase in firing rate, and consequently a resulting change to the functional nature of the cultures.

### Functional connectivity poorly correlated with intercellular distance

As previous literature has demonstrated strong functional relationships within individual brain regions, and negative correlations between connectivity and distance^51^, we investigated whether there would also be a similar relationship between cellular distance on the micron scale and functional connectivity in cultured networks. The Euclidian distance between cells in these experiments was determined to estimate the relationship between intercellular distance and connectivity. It was expected that on the cellular scale, there would be a decrease in neural connectivity with an increase in inter-cellular distance. The results demonstrate that although there is an overall negative trend in network connectivity with increased distance, quantified using the Pearson’s correlation coefficient, the effect was not as pronounced as was initially expected. Supplementary Figure S5, E shows that for all experimental conditions in these studies, the regression coefficient showed a value of −0.2847, indicative of a negative trend, but not a strong linear relationship. This relationship is maintained for each individual experimental condition (Supplementary Figure S5, A–D). This was strongest in the control and cells exposed to 100 µM glutamate, but still indicative of only a weak rather than a strong trend. This is further illustrated in Supplementary Figures S5, A–D, which highlight histograms of the Pearson’s coefficient between intercellular connectivity and distance, acquired for each culture. This is in contrast to Supplementary Figure S6, where the data is normalized by Z-scoring, and all the culture data is compiled in a given set of conditions together, further verifying the weak relationship between connectivity and distance. It is likely that there is not a strong relationship between connectivity and distance at these dimensional scales within the relatively small optical field-of-view (FOV), and that it would likely require larger intercellular distances to see notable effects. It is also known that myelination promotes more rapid propagation of neural signals across greater distances, and consequently functional connectivity would remain high despite the increased distance.

## Discussion

In summary, we have developed an algorithm and experimental method for identifying network connectivity in cultured neuronal networks. This algorithm overcomes the limitations imposed by other methods by using metrics for quantifying neural connections, directionality of signal propagation, and acquisition of firing parameters to understand bulk properties of the network. Experiments were performed that measured functional activity from neural cultures that expressed GCaMP6s—a GECI, under control conditions, and after chemical stimulation with glutamate. We were able to identify significant differences in network connectivity as well as in percent-change between control cultures and those exposed to the external chemical stimuli. Differences were also noted between cultures exposed to different levels of chemical stimuli, with significant differences between baseline measurements and those after chemical stimulation. Baseline measurements were used for quantifying the inherent firing and connectivity properties of the cultured networks, which provided a means for then quantifying causal changes and inherent variations, and how these properties subsequently changed after experimental stimuli. This algorithm and experimental technique, hence, serve as a useful tool for inferring network activity in cultured networks, demonstrating that we do not need to rely solely on network simulations to inform us of network connectivity. This algorithm further serves, among other algorithms, as an effective means for identifying network topology, and potentially as a method for understanding how neurons function to develop various behaviors.

Most interestingly, with optical imaging, the implications of time-varying analysis of network connectivity and a combined time-frequency approach could unveil many otherwise unexplored phenomena. Beyond the ability to infer directionality of signal propagation is the strength of identifying instances of significant connectivity changes, and identification of the frequency content imposed by specific stimuli. In the context of this work, a convergence of glutamate-evoked responses to roughly 0.3 Hz signifies that the receptors specific to glutamate cause neurons to fire at this frequency. Physiologically, these slow oscillations tend to be associated with delta waves, which are involved in non-rapid eye movement (NREM) and rapid-eye movement (REM) sleep^52^. Studies have shown that in the hippocampus, changes at these frequencies tend to be associated with disruptions to sleep^53^, which can be reduced by induction into deeper stages of sleep. Further studies suggest that glutamate and GABA concentrations correlate strongly with wakefulness, and increases in glutamate tend to be evoked by disruptions to sleep patterns^54^ and from deprivation. These studies each utilized EEG as the analytic method, and used for doing so, and used a time-frequency spectrogram to monitor frequency content at different sleep stages. This could also easily be done with optical microscopy setups to provide spatio-temporal information related to this type of stimulus, providing even stronger evidence of this.

The implications of using a joint-approach have also been demonstrated, as each of the outlined metrics identified complementary and supplementary information that may not necessarily be readily accessible through any individual metric. The inter-event intervals, for example, demonstrated an overall decrease in inter-event intervals for all experimental conditions, including the control. Although the effect was more pronounced at the higher concentration of glutamate, there was still a decrease in the control conditions. This can be attributed to the sensitivity of neurons to any change in their external environment. Although care was taken to pre-incubate all chemical reagents in a CO_2_ incubator, sudden changes in pH and osmotic conditions could immediately shock the cells, requiring time for the cells to equilibrate in a new environment due to their increased excitability under these conditions^55–58^. Although this did not significantly affect connectivity in comparison to the glutamate-treated samples, it did affect the firing rate of all cells. This did not necessarily result in simultaneous rapid firing, however, as the correlation coefficients did not change dramatically in all cases. So although there were some notable differences that were identified by one metric, these differences were not necessarily identified by the other metrics. This demonstrates the strength of measuring each of these parameters, as there were global changes to the culture that did not necessarily affect global connectivity after the addition of more cell culture media. Each of these metrics demonstrates the ability of the algorithm to not only monitor network properties and provide detailed information about the network, but also reveal connectivity at a single-cell level under different conditions.

The translation of these techniques to optical microscopy could further this knowledge, and also provide insights that have otherwise gone unexplored with traditional connectivity metrics, but on a smaller scale. The inherent limitation with microscopy is the limited FOV, which makes it difficult to retrieve information transfer between more distributed neural circuits across more distant brain regions. Combining traditional microscopy and the proposed computational methods to meso-scale microscopy^20^, however, would help overcome this limitation. Multimodal microscopy could also be used to investigate metabolic changes imposed by this sort of stimulus^59,60^, and how it relates to the calcium information brought by GCaMP imaging. Coupling this with electrophysiology would also provide further verification of the type of signals being accessed, and how this relates to the underlying signaling pathways unveiled through optical imaging. Microscopic imaging of neural systems, hence, would benefit significantly from the breadth of information provided by the proposed algorithm, facilitating the discovery of complex neural phenomena.

With the combination of optogenetics and optical imaging, this algorithm could be used to guide and interrogate neural tissue in a more directed and informative way. We propose that this algorithm can accomplish this inherently through the use of wavelet coherence analysis by acquiring statistics about phase differences and phase stability of these signals between cells over time. Single cell excitation and the subsequent measurement of phase delays between multiple active neurons, coupled with this algorithm, would identify these direct connections in a way that other connectivity approaches cannot. If there were a consistent phase delay between two signals during a certain behavior or stimulus, this approach would directly measure this across different signal frequencies, and be used to infer causality. Smaller phase differences would generally be attributable to more direct signal propagation. By identifying the predominant frequencies within these signals, as was investigated in this study, and by obtaining the phase delay at these frequencies, this data would be readily utilized by the wavelet coherence approach. This information could then be used to drive weighted optical excitation of neural tissue, where the more direct connections are identified and perturbed for efficient elicitation of activity, while other active but less prominent connections are minimally excited or omitted from excitation. Adoption of these transient connectivity measures would thus facilitate investigation of connectomic studies to obtain information otherwise inaccessible with other approaches. Beyond the context of these studies, the algorithm and approach could also be beneficial for comprehensively understanding the effects of drugs on neural cultures, tissues, and other samples. Their effects on firing frequency, functional connectivity, firing rate, directionality, and other factors outlined in this work can be tracked over time. This could also be utilized for monitoring the effects of different disease models on neural communication and function, and the effects of different treatments on regulating these effects. This comprehensive analytical framework is a useful tool that can be widely implemented in optical microscopy and potentially across larger spatial scales. Further advancements of time-varying analyses to inherently time-variant neural mechanisms would no doubt foster knowledge about the temporal nature of these complex systems, and coupled with advances optical imaging, provide extensive information about the molecular underpinnings of neural function.

## Methods

### Algorithm

The algorithm framework is illustrated in Fig. 1, which starts by loading the calcium imaging videos into the Matlab workspace for analysis. Upon loading the sequence, cells of interest were segmented by manual region of interest (ROI) selection from the temporal projection of the mean of the sequence, specifically targeting the soma (Supplementary Figure S1). The fluorescent activity from the identified cells was then extracted. The raw fluorescence transients were used to calculate change in fluorescence, or $\frac{\triangle F}{{F}_{0}}$ transients. A 5-pixel location from each neuron soma was selected, followed by a region with no observable fluorescence. This fluorescence-free region represented the background noise, which was superimposed on all signals. These 5-pixel areas were averaged at each instance of time, and these averages were used as the representative signal from each cell, and the background. The 5-pixel background was then subtracted from the fluorescence from each individual cell. Baseline fluorescence was identified as the average, minimum fluorescence from individual cell bodies, denoted as ${F}_{0}$ in this work. The term $\triangle F$ is defined as the change in fluorescence after an interval in time, after subtracting ${F}_{0}$.

Upon extraction of these transients, a temporal correlation coefficient for the identified time window was calculated to identify linear relationships between identified cells. Similarly, the power spectrum was calculated for the $\frac{\triangle F}{{F}_{0}}$ transient of each cell, and a correlation coefficient was obtained between cells to compare the spectral similarities across the calcium transients for these cells. However, this provided only a holistic view of the transients, and ignored the minutiae of fluorescence activity between neighboring cells. To more definitively acquire this information, the image sequence was divided into six smaller time-windows (ten second width), and a correlation coefficient was calculated within each smaller window. Thereafter, these coefficients were averaged for each cell, and used as a correlation coefficient for each larger, one-minute time window. Finally, the derivative of each trace was used as an assessment of functional connectivity as well. Some action potential inference algorithms use a derivative method for inferring when action potentials occur^61–63^, due to the increased signal prominence that comes about as a result of this. This work uses the derivative of calcium transients to identify regions of significant activity, and consequently, as a metric for electrical activity. The derivative for each trace was obtained by first applying a smoothening filter to the data using an unweighted, 2^nd^ order least-squares averaging model with 2.5% span (“smooth” operator in Matlab, using “rloess” method). Thereafter, the derivative was taken across the smoothened filter data, and the correlation coefficient between cells was calculated for the filtered data. These four coefficients were then averaged, and used as the connectivity coefficients, or weights, associated between cells. These were averaged to incorporate similarities from each metric of interest to contribute to the final, functional synaptic weight between cells. The magnitude varies due to differences in each of these metrics, so each was equally weighted in the final metric to ensure there was not one that was more influential over the others. Each individual metric allows for comparing the similarities of different aspects of the signals of interest between cells, and consequently their connectivity. In this work, coefficients between 0 and 0.3 are empirically treated as having weak correlation between two variables, 0.3 to 0.5 as low correlations, 0.5 to 0.7 as moderate, 0.7 to 0.9 as strong, and coefficients above 0.9 as very strong correlations^64^. Furthermore, connectivity between cells in this work is assumed to arise from changes in connectivity across synaptic-dendritic interfaces, rather than changes in conductivity along axons or dendrites. This is due to the increased excitability of neurons in response to external glutamate, rather than due to changes at the synaptic cleft^65^.

Furthermore, the proposed algorithm performs this process over the course of larger, 1-minute intervals. As per the experimental design, each culture was imaged for a period of ten minutes. To assess network connectivity and connectivity variance over time, the correlation coefficient was calculated for each 1-minute interval of the video sequence, and compared over time. To compare average network activity between experiments, the coefficients were averaged across all ten, 1-minute intervals. Finer coefficients were calculated at ten-second intervals for the purposes of tracking temporal connectivity dynamics, whereas the larger one-minute intervals were used for the overall coefficient to have a significant number of data points to represent overall connectivity. Variations in connectivity were also assessed by calculating the percent-change of each one-minute interval in a ten-minute video, and averaging these percent-changes across this ten-minute time-frame. This algorithm provides a method for assessing the weights between cells based on their functional activity under various experimental conditions.

Signals were further analyzed to acquire the timing of individual cellular events over time. After calculating the $\frac{\triangle F}{{F}_{0}}$ for each cell and applying a 3 Hz, low-pass, third-order Butterworth filter for noise reduction, significant calcium events were identified by thresholding. The threshold was determined by calculating the mean (µ) and standard deviation (σ) for a given calcium transient, and setting the threshold to µ + σ. The “findpeaks” function in Matlab was then used for peak detection using this threshold. Peaks were also isolated if their widths were at least 0.5 seconds (due to the average duration of calcium events being nearly 0.5 seconds), and if neighboring peaks were separated by no less than 0.5 seconds. These metrics ensured that only significant peaks above the mean fluorescence were used to separate peaks from noise, and to further ensure this by not counting two local peaks from the same event. The temporal location of each of these peaks was then stored in memory. The time between any two significant events for a single calcium transient, called the inter-event intervals, were also calculated. Previous work has shown that the firing patterns of neurons, in many cases, follow a Poisson process^66,67^, establishing the inter-event interval metric as a specific property of a neuronal network under different conditions.

Finally, wavelet analyses were employed to measure the dynamic nature of cellular communication, and establish the degree to which cells may be inter-correlated over time. The wavelet transform is a standalone method for measuring the frequency content of signals continuously over time^41^. As such, any perturbations to signals as a result of some external stimulus can be tracked and quantified. This has been performed in this study for individual cells, and for the mean signal properties. Complementary to the time-varying connectivity approach, the wavelet-coherence approach provides a near-continuous metric to show temporal fluctuations in the degree of coherence between two signals^49^. In contrast to other metrics, it also provides directionality of information flow using the phase difference between two signals throughout the spectrum of sampled frequencies^49^. If the signal came from one cell at the prominent signal frequency, this would be identified. The ability to have this information over time also allows for the mean and variance of these signals to be quantified, establishing the degree to which two cells may be inter-correlated over time. The wavelet approach, thus, encompasses the benefits of many other metrics, and provides a powerful avenue for assessing network properties and dynamics. In this work, the coherence and phase matrices are generated and compared for pairs of cells over time. Additionally, the wavelet transform was implemented to track the prominent frequencies at various points in time.

### Sample preparation

All animal experiments were conducted in accordance with the relevant guidelines and regulations in a protocol approved by the Institutional Animal Care and Use Committee (IACUC) at the University of Illinois at Urbana-Champaign. Transgenic mice (GCaMP6s, Jackson Labs) were sacrificed at days 2 or 3 postnatal (P2-P3) to harvest hippocampal tissue. Tissue was stored in Hibernate-A (HA) and used the following day for culture. All procedures were carried out under sterile conditions in a biosafety cabinet (BSC). Imaging dishes were prepared the day of culture by coating with 60 µL of poly-D-lysine (PDL), and incubating at room temperature for a minimum of two hours. After this, dishes were washed with sterile DI water and left to dry for at least one hour. The hippocampal tissue was washed twice with 10 mL of HA, and subsequently agitated with a HA-Calcium/papain mixture for 25 minutes. The tissue was then mechanically dissociated by aspirating twice with a 20 G needle and repeated once more with a 22 G needle. The dissociated cells were isolated using a 20 μm filter, and then centrifuged at 2000 rpm for 6 minutes. The supernatant was removed with a vacuum, and the cells were suspended in 500 μL of DMEM. Upon suspension, the cells were counted using a hemacytometer, and plated onto the coverslips to a final density of 200,000 cells per dish. These cells were then placed in a cell culture incubator (5% CO_2_, 5% humidity) for an hour. Finally, 500 μL of pre-incubated DMEM and 1 mL of NBActiv4 were added to the culture. Cultures were grown to DIV (days *in vitro*) 12–15, and media was changed with NBActiv4 every 3–4 days.

### Experimental setup and workflow

In this study, glutamic acid (glutamate) was used as a chemical stimulus to evoke cellular responses and induce a change in network connectivity. Glutamate is known to promote increases in neural firing^68^, and is an excitatory neurotransmitter associated with an increase in synaptic connectivity associated with depression^69,70^, memory encoding^71^, beta-amyloid associated functional connectivity^72^, and overall functional connections^68,73^. Hence, it was assumed that its introduction would result in increased network connectivity. To test the reliability of the algorithm for detecting network changes, hippocampal neuron cultures were placed in an incubation chamber (DH-35iL, Warner Instruments), which was adjusted to 33 °C–35 °C, and placed under a microscope (Axio Observer D1, Zeiss) with a 20x objective. A broadband white light source (X-Cite, Lumen Dynamics) was used for illumination. The GCaMP6s signal was isolated using a stock dichroic mirror (495 nm) and GFP filter (525 ± 25 nm) to allow for excitation and emission. The cultures were imaged using a monochrome CCD camera (AxioCam 503, Zeiss) at 22.7 Hz, controlled with commercial software (Zen PRO, Zeiss). After the initial imaging session, various concentrations of glutamate (10 µM, 25 µM, and 100 µM) were perfused into the incubator chamber using a pre-incubated, 1 cc syringe, while the cells were imaged. Control samples were perfused with 250 μL of NBActiv4 cell culture medium. A schematic of the experimental procedure is shown in Supplementary Figure S1.

### Analysis

The algorithm was run across experimental data using custom Matlab code. The correlation coefficients between experiments were compared using a separate script for statistical analysis. A two-tailed Welch’s t-test was used to determine what statistical differences existed pre- and post-excitation with glutamate. Coefficients were also compared between the control samples for statistically significant differences. Correlation coefficients were Z-scored to normalize the data appropriately when compared between experimental conditions and cultures.

## Supplementary information

Supplementary Information.

## Data Availability

The data and code that support these results are available from the corresponding author upon reasonable request and through collaborative investigations.
