# Supplementary material for: Dynamic Tracking Algorithm for Time-Varying Neuronal Network Connectivity using Wide-Field Optical Image Video Sequences
Source: Sci Rep. 2020 Feb 13;10:2540. doi: 10.1038/s41598-020-59227-5 (PMC7018813; doi:10.1038/s41598-020-59227-5)
Supplement: Supplementary file 1 — Supplementary Information. [file 41598_2020_59227_MOESM1_ESM.pdf]

# Dynamic Tracking Algorithm for Time-Varying Neuronal Network Connectivity using Wide-Field Optical Image Video Sequences

Carlos Renteria[1,2], Yuan-Zhi Liu[1], Eric J. Chaney[1], Ronit Barkalifa[1], Parijat Sengupta[1], Stephen A. Boppart[1,2,3,4,5,*]

[1]Beckman Institute for Advanced Science and Technology
[2]Department of Bioengineering
[3]Department of Electrical and Computer Engineering
[4]Neuroscience Program
[5]Carle Illinois College of Medicine
University of Illinois at Urbana-Champaign

*Corresponding Author: boppart@illinois.edu

**Supplementary Information:**

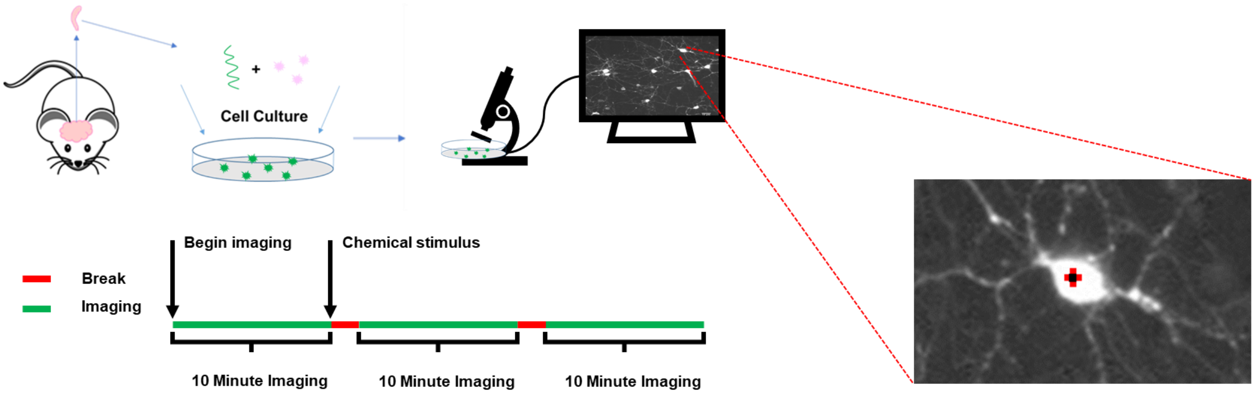


**Figure S1:** Experimental design for imaging and the glutamate stimulus. Schematic illustrates hippocampal isolation from P2-P3 pups, their culture, and placement on the microscope stage for imaging. For the glutamate application timeline, green regions indicate time periods of imaging, and red regions indicate when imaging was suspended. The crosshair used to isolate the fluorescence activity from an identified cell body is shown on the far right. The black region on the crosshair denotes the point of selection on a neuron cell body and the centroid, and the surrounding red pixels represent the other regions used for averaging fluorescent activity.

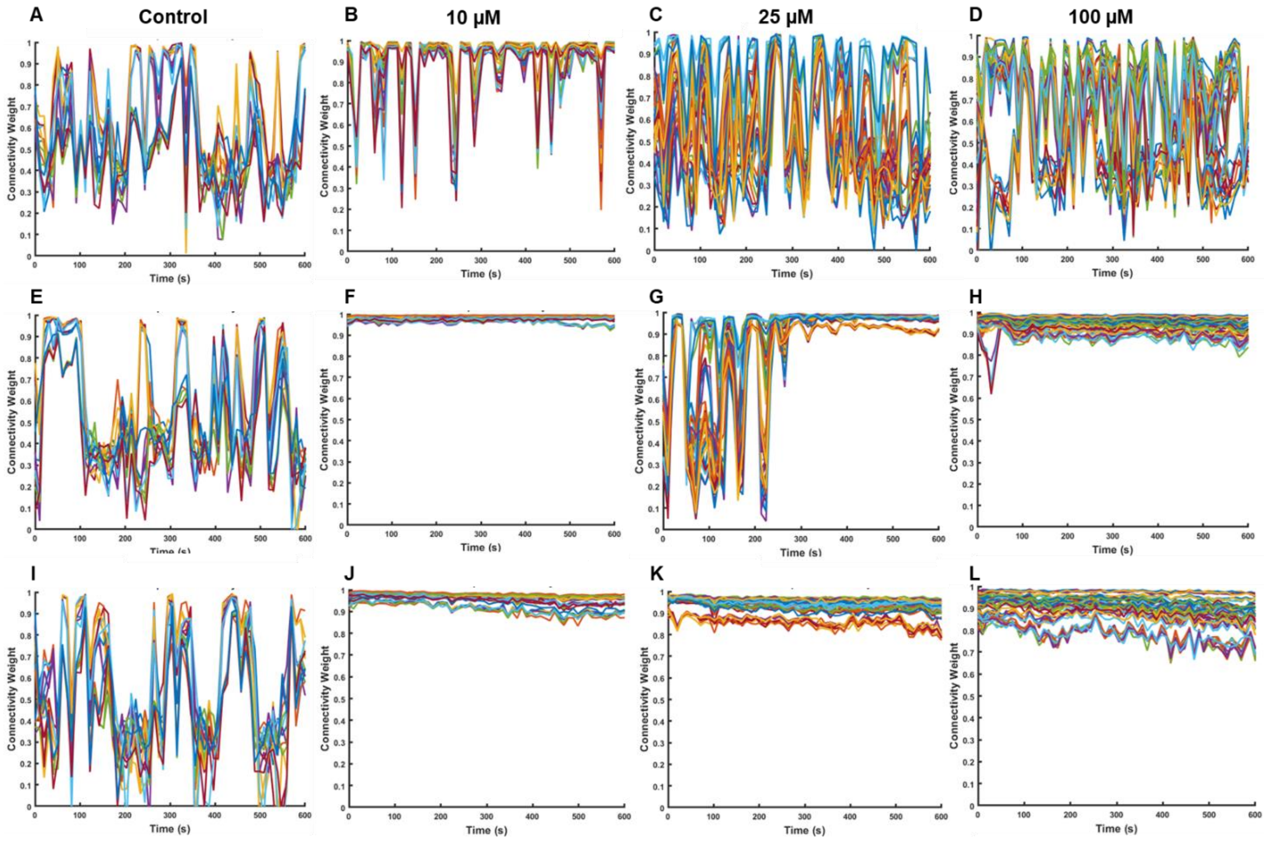


**Figure S2:** Time-varying connectivity plots of a control, 10 µM, 25 µM, and 100 µM glutamate stimulated cultures, over each ten-minute imaging period. Plots show the time-varying connectivity between all pairs of cells (A-D) before the application of chemical stimulus, and (E-H) 10 minutes and (I-L) 20 minutes after chemical induction.

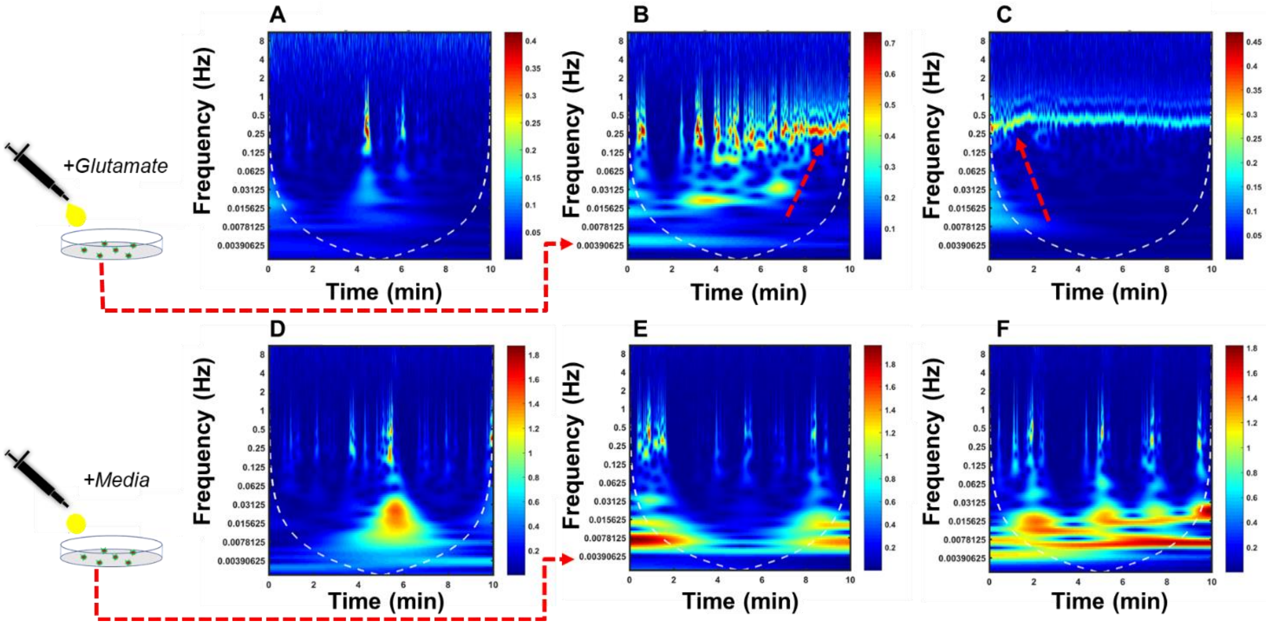


**Figure S3:** Representative wavelet-transforms from individual cells (A, D) before and then after the application of (B, C) a chemical stimulus (25 µM glutamate) and (E, F) control solution (culture media).

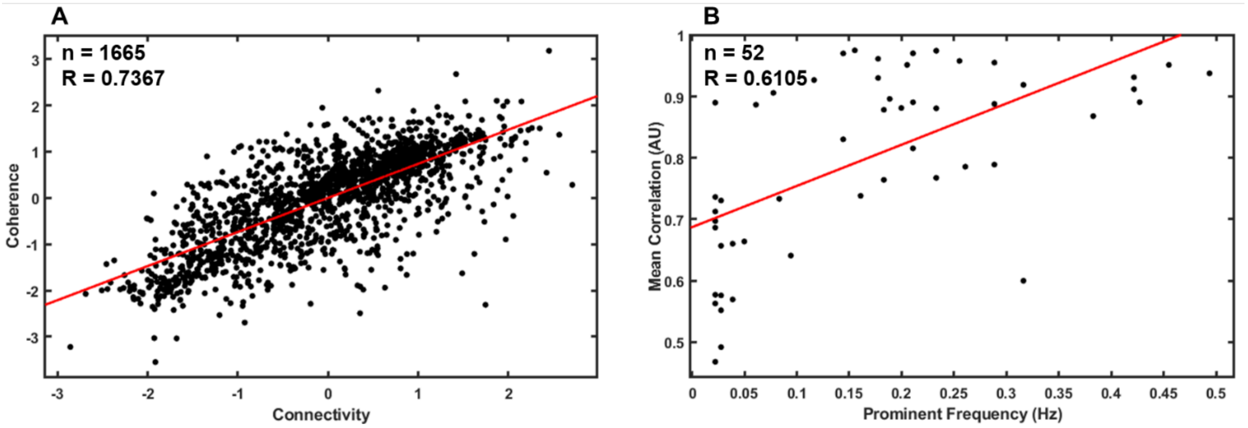


**Figure S4:** Regression coefficients between the normalized coherence and connectivity values for all experimental conditions (A), and between prominent cell frequency and connectivity (B). Each pair (n=1665) of cells in an experimental condition for coherence has a corresponding connectivity and coherence value, so these pairs were matched, normalized, and regressed to achieve the correlation coefficient of 0.7367. There is also a strong linear correlation between measured connectivity and increased firing frequency in cell cultures (B), with a Pearson's coefficient of 0.6105 between both variables, for all cultures utilized in this study (n=52).

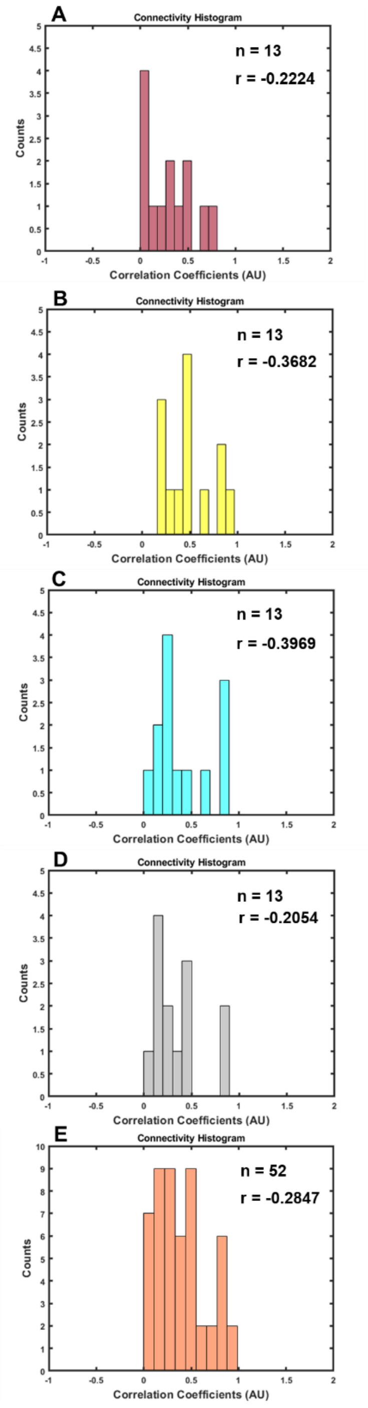


**Figure S5:** Histograms and probability density functions (PDFs) for cellular connectivity and their relation to spatial proximity for (A) control, (B) 10 µM, (C) 25 µM, and (D) 100 µM glutamate concentrations, and (E) all conditions combined. The relationships are generally normally distributed, with a mean centered around 0.4. Sample size: n = 52 imaging sessions. Sample size: n = 13 imaging sessions for each.

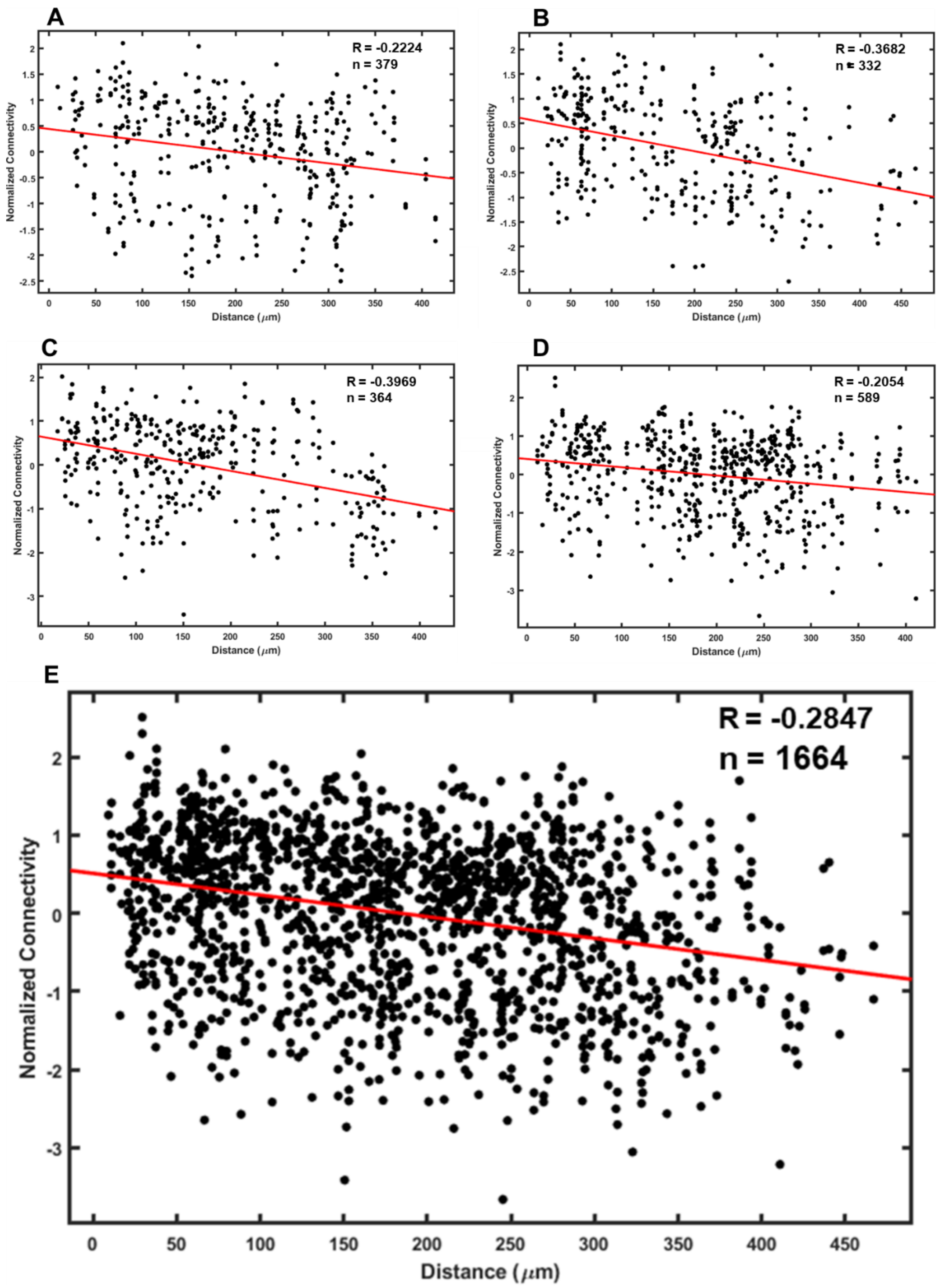


**Figure S6:** Regression coefficients between the normalized connectivity coefficients and the intercellular distance for (A) the control, and for (B) 10 µM, (C) 25 µM, and (D) 100 µM conditions. (E) Regression coefficients for all conditions combined between the connectivity coefficients and the inter-neuron distance, with weak correlations detected for all conditions.
